# Supplementary material for: Novel feature extraction method for signal analysis based on independent component analysis and wavelet transform
Source: PLoS One. 2021 Dec 16;16(12):e0260764. doi: 10.1371/journal.pone.0260764 (PMC8675669; doi:10.1371/journal.pone.0260764)
Supplement: Attachment — Submitted filename: Reviewers comments _Nedal Al Taradeh.pdf [file pone.0260764.s001.pdf]

The contribution of this paper is exciting and original. The proposed method seems to be valid for signal feature extraction. The procedure using signal processing can successfully lead to separation between the signal waveform from its resources. Experiments were carried out with an MIT-BIH database to illustrate the four classifications(Normal, Vestibular ectopic beats, Ventricular ectopic beats, and Fusion strikes). The derivations, experimental setup, and discussion section are clear to the readers. I think this paper will exhibit a better understanding of ECG heartbeat classification. Therefore, I recommend accepting this manuscript as it is with some polishing comments:

C1. The abstract shall be rewritten to reflect all results yielded.

C2. The Introduction could be shorter with more concentration on state of the art and the advantage of the proposed methodology.

C3: In any case, profound grammatical revisions are needed to render the document useful for readers.

C4: The article should have clear arguments regarding the proposed method's rationale and motivations and explicit discussions of the advances versus the prior art.
